# Illinois State Veterinary Medical Association

**Published:** 1893-10

**Authors:** Matthew Wilson

**Affiliations:** Sec’y.; Mendota, Ill.


*Secretary.*

---

*Illinois State Veterinary Medical Association.*—The semi-annual meeting of the Illinois State Veterinary Medical Association was held at Peoria, Ill., Feb. 17th, '93. The meeting was called to order by Pres. S. S. Baker. The roll being called the following members responded to their names :

Drs. A. G. Alverson, S. S. Baker, G. Z. Barnes, G. W. Browning, George Ditewig, C. E. Hollingsworth, C. D. Hartman, J. T. Nattress, J. W. Parkinson, Jno. Scott, N. I. Stringer, H. Thompson, M. Wilson.

Minutes of the previous meeting were read and approved.

Committee on Legislation reported through its chairman, Dr. Baker, on the work done in drafting a bill to be presented to the Legislature, and the sending of letters and petitions to representative live stock men in this and surrounding States and receiving their indorsement to such bill.

Committee on Form of Certificate of Membership was given power to act in selecting and having printed an appropriate certificate.

The following gentlemen's names were proposed for membership: Dr. W. S. Wingate, "Ch. '92," Farmington, Ill.; Dr. R. P. Steddom, "Ont. '86," Galesburg, Ill.

On motion of Dr. Stringer, seconded by Dr. Hollingsworth, the rules were suspended for the time being and the above-named gentlemen elected by acclamation.

It was moved by Dr. Scott, seconded by Dr. Barnes, that the Secretary be instructed to notify all members in arrears asking for their dues. Motion carried.

AFTERNOON SESSION.

Meeting was called to order at 2:00 P. M.

Dr. W. W. Giles, "Ch. '92," Eureka, Ill., was proposed for membershp and elected by acclamation.

Dr. Ditewig then read his paper on "Remarkable Cases in Practice" Discussion closed on motion.

Dr. Stringer then read his paper on "Strongylus Tetracanthus." Discussion was closed on motion.

Dr. Thompson then presented a paper on "Case Report," discussion of which was closed on motion.

Dr. Scott was then called on for his paper on "Castration," and was followed by Dr. S. S. Baker on "Typhoid Fever in the Horse."

Meeting adjourned until after supper.

Members called to order at 7.30 P. M.

After some further discussion of Dr. Baker's paper on "Typhoid Fever in the Horse," a vote of thanks to the essayists was proposed by Dr. McConnell, and responded to by Drs. Baker and Stringer.

A vote of thanks was also presented the proprietors of the hotel for their accommodations, and the meeting adjourned to come together at the call of the committee in Chicago next November.

MATTHEW WILSON, M. R. C. V. S., *Sec'y.*

MENDOTA, ILL.

